# THE EFFECTIVENESS OF A VIRTUAL INTERGENERATIONAL ACTIVITY FOR REDUCING YOUNGER AND OLDER ADULTS’ AGEISM

**DOI:** 10.1093/geroni/igad104.1164

**Published:** 2023-12-21

**Authors:** Janelle Fassi

**Affiliations:** University of Massachusetts Boston, Boston, Massachusetts, United States

## Abstract

This presentation will discuss results from a study that examined whether a virtual intergenerational activity was feasible and effective for reducing ageism among older (OA) and younger adults (YA) during the COVID-19 pandemic. OA (n = 16) and YA (n = 15) completed a virtual intergenerational activity, and analysis was conducted for a subsample (n = 5 OA, n = 10 YA) who completed pretest-posttest ageism surveys. Paired samples t-tests showed no significant ageism change in attitudes toward OA. OAs’ ageist attitudes toward YA (p = .012) were significantly reduced. Open-ended responses revealed several themes (e.g., challenging ageist stereotypes), which contrasted with statistical findings among the YA sample. Preliminary evidence demonstrated the feasibility of this activity. Further, OA and YA gained greater respect and understanding for each other as indicated by the open-ended responses. However, a larger sample size is needed to better determine efficacy. Nonetheless, the key takeaways from this presentation will help educators and program organizers alike explore when and how a virtual intergenerational activity can bridge gaps between older and younger adults.

